# Studies on the Alkaloids of the Calycanthaceae and Their Syntheses

**DOI:** 10.3390/molecules20046715

**Published:** 2015-04-15

**Authors:** Jin-Biao Xu, Ke-Jun Cheng

**Affiliations:** Chemical Biology Center, Lishui Institute of Agricultural Sciences, 827 Liyang Street, Lishui 323000, Zhejiang, China; E-Mail: xujinbiao2015@126.com

**Keywords:** biological activity, biosynthesis, Calycanthaceae alkaloids, structure, synthesis

## Abstract

Plants of the Calycanthaceae family, which possesses four genera and about 15 species, are mainly distributed in China, North America and Australia. Chemical studies on the Calycanthaceae have led to the discovery of about 14 alkaloids of different skeletons, including dimeric piperidinoquinoline, dimeric pyrrolidinoindoline and/or trimeric pyrrolidinoindolines, which exhibit significant anti-convulsant, anti-fungal, anti-viral analgesic, anti-tumor, and anti-melanogenesis activities. As some of complex tryptamine-derived alkaloids exhibit promising biological activities, the syntheses of these alkaloids have also been a topic of interest in synthetic chemistry during the last decades. This review will focus on the structures and total syntheses of these alkaloids.

## 1. Introduction

The small family of the Calycanthaceae comprises four genera, namely *Chimonanthus* Lindley, *Sinocalycanthus* Cheng & S. Y. Chang, *Calycanthus* L., and *Idiospermum*, which globally include *ca*. 15 species [1,2,3]. The plants of the *Chimonanthus* and *Sinocalycanthus* genera are ornamental shrubs endemically distributed in China, and those of the *Calycanthus* and *Idiospermum* originate from North America and Australia, respectively. The classification of the species of *Chimonanthus* genus is still a tough task and has been a subject of debate for a long time [4]. The early literature categorized this genus into three species, *i.e.*, *Ch. nitens* Oliv., *Ch. praecox* (Linn.) Link, and *Ch. salicifolius* S.Y. Hu [5]. Recently it was proposed that this genus be classified in 10 species based on morphological evidence, including *Ch. nitens* Oliv., *Ch. praecox* (Linn.) Link, *Ch. salicifolius* S.Y. Hu, *Ch. nitens*, *Ch. zhejiangensis* M.C. Liu, *Ch. campanulatus*, *Ch. baokangensis*, *Ch. anhuiensis*, *Ch. caespitosa*, *Ch. campanulatus* var. guizhouensis [6]. In addition, only one species, *C. chinensis* Cheng et S.Y. Chang, is attributed to the *Sinocalycanthus* genus. Three plants named *C. floridus* var. floridus, *C. floridus* var. laevigatus, and *C. occidentalis* Hook. et Arn. pertain to the genus *Calycanthus*. The plant *Idiospermum australiense* (Diels) S. T. Blake a rare tree that occurs only in the North Queensland region of Australia is the sole member of the *Idiospermum* genus. These Calycanthaceae plants are primitive angiosperms and popular ornamental flowers with a pleasant aroma. The Calycanthaceae plants have long been used in Traditional Chinese Medicines (TCMs), to treat rheumatic arthritis, coughs, throat wounds, dizziness, nausea, fever, detoxification, and enteral disease [7,8,9].

**Figure 1:**
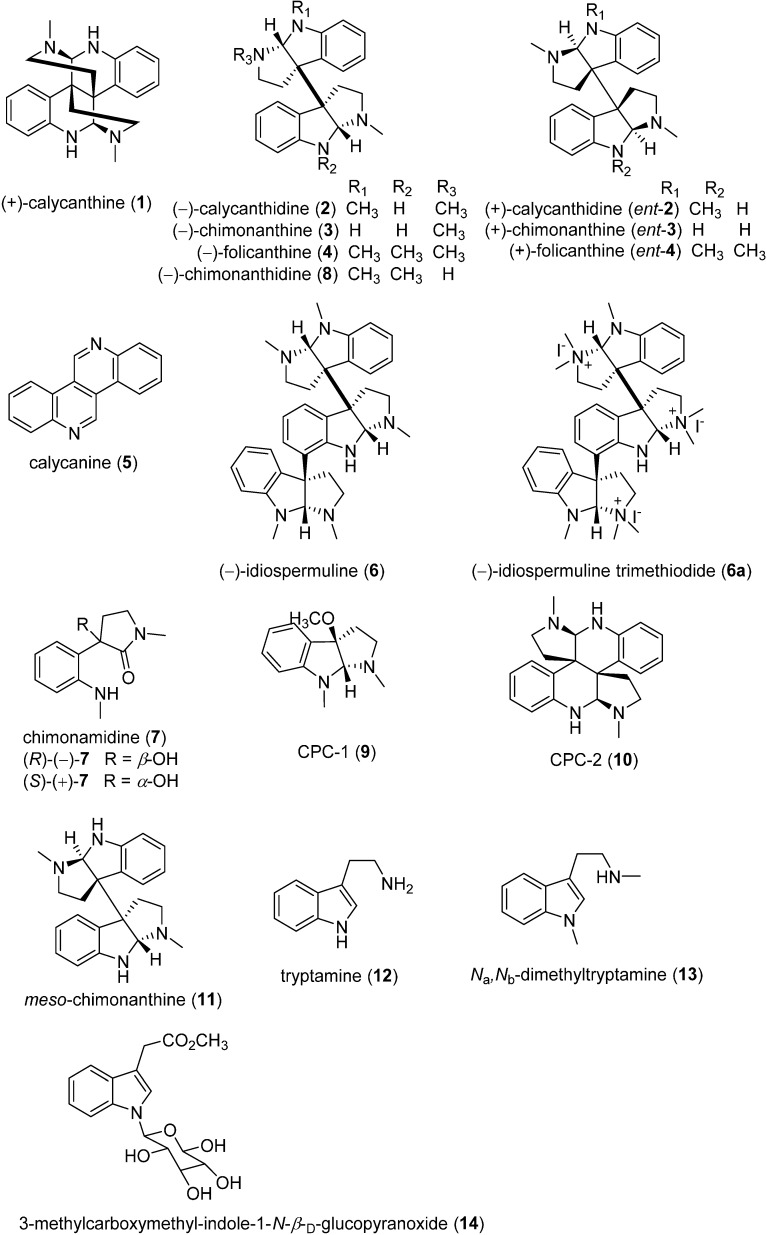
Structures of the Calycanthaceae alkaloids.

The chemical investigation of the Calycanthaceae plants started more than one hundred years ago in 1888, which led to the isolation of a large amount of alkaloids, flavonoids [10], lignans [6,11], coumarins [12,13,14], terpenoids [15,16,17], and essential oils [7,18,19,20]. It is important to note that the discovery of the Calycanthaceae alkaloids was reminiscent of the history of development of science. Looking back to this history, only 14 alkaloids (Figure 1) whose discoveries were full of hardship and arduousness were characterized from this family. During past decades, there has been a trend towards e synthetic approaches of these structurally interesting and bioactive alkaloids. This review attempts to provide timely and comprehensive coverage of the chemical and biological studies related to the Calycanthaceae alkaloids, with a specific focus on summarizing the great amount of synthetic work performed in this area.

## 2. Structures, Biological Activities, and Biosynthetic Origins of Calycanthaceae Alkaloids

### 2.1. Structures of Calycanthaceae Alkaloids and Their Discovery

The phytochemical investigation of the plants Calycanthaceae was first described in 1888, which led to isolation of the first Calycanthaceae alkaloid, (+)-calycanthine (**1**, Figure 1) with a dimeric piperidinoquinoline skeleton, from the seeds of *C. glaucus* Willd. by Eccles. One year later, Wiley proved the high content of this alkaloid in the seeds of the same plant [21]. In 1905, further progress was made by Gordin whereby this principal alkaloid was crystalized in different forms at ordinary temperature and deduced to possess a molecular formula C_11_H_14_N_2_ containing no oxygen atom [21,22]. A few years later, Späth and Stroh expressed their disagreement on Gordin’s work and stated that the empirical formula of (+)-calycanthine should be doubled C_11_H_14_N_2_ [23]. Soon Manske put forward the possibility that this molecular formula might be C_22_H_26_N_4_ [24], which was finally proved by Barger’s group in 1939 [25]. The structure of this alkaloid (+)-calycanthine, C_22_H_26_N_4_, was finally established uniequivocally by means of X-ray crystal structural analysis of its dihydrobromide dehydrate by Hamor’s group in 1960 [26].

In 1905, Gordin also reported a second alkaloid, isocalycanthine, from the seeds of *Chimonanthus* genus, which possessed a different melting point and showed different behavior with respect to the removal of water of crystallization from the hydrated base with those of calycanthine [27,28]. However, Manske expressed doubts about the existence of isocalycanthine. In his study on the species of *C. floridus* L., a great quantity (1.2%) of calycanthine was. Gordin’s seed extract consisted in reality of *C. fertilis*. In the same report, Gordin also discribed the isolation process of (+)-calycanthine (2.6%) from the seeds of *Meratia praecox* (*C. praecox*) [24]. In 1992, the occurrence of isocalycanthine in a closely related species *Psychotria forsteriana* (Rubiaceae) was reported by Kuballa’s group [29].

In 1938, Barger’s group isolated a minor alkaloid, (−)-calycanthidine (**2**), from the seeds of *C. floridus*, which molecular formula was deduced to be C_13_H_16_N_2_ [30]. This conclusion was corrected by Saxton in 1962, who established both the formula C_23_H_28_N_4_ and the structure of (−)-calycanthidine [31]. The alkaloid (−)-chimonanthine (**3**) was firstly obtained from *Ch. fragrans*. Lindle (*Ch. praecox*) by Hodson’s group [32], and its structure was determined on the basis of detailed X-ray analysis of chimonanthine dihydrobromide by Grant’s group [33,34]. As for (−)-folicanthine (**4**), it was first isolated by Hodson’s group in 1957 and was identified to be a dimeric pyrrolidinoindoline alkaloid, close to (−)-calycanthidine (**2**), and (−)-chimonanthine (**3**) [32]. From then on, this seemed to be an end for the structural elucidation of (−)-calycanthidine, (−)-chimonanthine, (−)-folicanthine, until in 2000, the total syntheses of these alkaloids were completed by Overman’s group, proving that (−)-calycanthidine, (−)-chimonanthine, (−)-folicanthine should be drawn as compounds **2**, **3**, and **4** (Figure 1), respectively; therefore the structures of compounds *ent*-**2**, *ent*-**3**, and *ent*-**4** reported in the previous papers should be (+)-calycanthidine, (+)-chimonanthine, (+)-folicanthine, respectively [35]. Calycanine (**5**) with a molecular formula C_16_H_10_N_2_ was a product of calycanthine and chimonanthine obtained by Zn dehydrogenation. Its structure was first incorrectly proposed by Barger, and then revised by Woodward’s group via synthesis [32,34,36].

(−)-Idiospermuline (**6**), a trimeric pyrrolidinoindoline, together with two known dimeric alkaloids, (+)-calycanthine and (−)-chimonanthine were isolated by the bioassay-guided method from the seeds of *Idiospermum australiense* (Diels) S.T. Blake, a native species from North Queensland, Australia. The structure of (−)-idiospermuline was determined by NMR and MS data and the absolute stereochemistry was established by X-ray crystallographic study of idiospermuline trimethiodide (**6a**) [37].

In 2004, Takayama’s group investigated the alkaloidal constituents of the seeds of *Ch. praecox* L., leading to the isolation of two new tryptamine-related alkaloids, chimonamidine (**7**) and chimonanthidine (**8**), together with the known (+)-calycanthine, (−)-chimonanthine, (−)-folicanthine, and (−)-calycanthidine. They conducted the total synthesis of (±)-chimonamidine to establish its absolute structure. As a result, natural chimonamidine showed optical rotation ${\left[\alpha \right]}_{D}^{19}$ = −12.6, which was significantly different with those of (*R*)-(−)-chimonamidine (${\left[\alpha \right]}_{D}^{23}$ = −178) and (*S*)-(+)-chimonamidine (${\left[\alpha \right]}_{D}^{23}$ = +171), suggesting that natural chimonamidine is a mixture slightly enriched with the (*R*)-(−)-enantiomer. Chimonanthidine (**8**) was determined to be *N*_b_-monodemethylfolicanthine by total synthesis, with the absolute configuration eventually confirmed by a combined strategy of comparing the CD spectrum with that of known (−)-folicanthine [38]. In his paper of 2006, a further phytochemical study on the seeds and rinds of *Ch. praecox* (L.) f. concolor resulted in the isolation of a new pyrrolidinoindoline-type alkaloid, CPC-1 (**9**), and one new tetrahydroquinoline dimeric alkaloid, CPC-2 (**10**), together with eight known alkaloids, (+)-calycanthine, (−)-chimonanthine, (−)-folicanthine, (−)-calycanthidine, (−)-chimonanthidine, *meso*-chimonanthine (**11**), tryptamine (**12**), and *N*_a_,*N*_b_-dimethyltryptamine (**13**) [39].

In 2009, an antifungal activity-guided phytochemical investigation on the defatted seeds of *Ch. praecox*, a species grown in Shaanxi Province of China, afforded two dimeric alkaloids, (+)-calycanthine and (−)-folicanthine [40]. Additionally, an additional indole-derived glycoside, 3-methylcarboxymethyl-indole-1-*N*-β-d-glucopyranoside (**14**), and the four known compounds (+)-calycanthine, (−)-chimonanthine, (−)-folicanthine, (−)-calycanthidine were obtained from the fruits and leaves of *C. praecox* in 2011 [15]. A recent phytochemical study on the flower buds of *Ch. praecox* also led to the isolation of five known dimeric alkaloids and several other compounds [41].

### 2.2. Biological Activities

The Calycanthaceae plants have been used as Traditional Chinese Medicines (TCMs) for the treatment of colds, and as sedative, antitussive, anti-hypertension, antioxidation, anti-inflammatory, and antitumor medicines [14,15]. As the important components of these plants, the Calycanthaceae alkaloids showed biological activities such as anti-convulsant, anti-fungal, anti-viral, analgesic, anti-tumor, and melanogenesis inhibitory properties.

The main representative alkaloid, calycanthine (**1**), has been recognized as a powerful centrally acting anti-convulsant for a long time [42,43]. It was reported that calycanthine may mediate its convulsant action predominantly by inhibiting of the inhibitory neurotransmitter GABA as a result of interactions with l-type Ca^2+^ channels and by inhibiting GABA-mediated chloride currents at GABA_A_ receptors [44].

(+)-Calycanthine (**1**) and (−)-folicanthine (**4**) were evaluated for their antifungal activities against five plant pathogenic fungi, *Exserohilum turcicum*, *Bipolaris maydis*, *Alternaria solani*, *Sclerotinia sderotiorum*, and *Fusarium oxysportium*. It turned out to be that *B. maydis* was the most susceptible to **1** with an EC_50_ value of 29.3 μg/mL, and then *S. sderotiorum* to **4** with an EC_50_ of 61.2 μg/mL [40]. (−)-Chimonanthine (**3**) and (−)-folicanthine (**4**) also showed weak antiviral activities against porcine respiratory and reproductive syndrome virus (PRRSV) with IC_50_ values of 68.9 ± 3.1 μM and 58.9 ± 10.2 μM, respectively [45].

Chimonanthines were tested on μ- and κ-opioid binding assay, and on the tail-flick and the capsaicin-induced pain models. As a result, (−)-chimonanthine (**3**), (+)-chimonanthine (*ent*-**3**), and *meso*-chimonanthine (**11**) showed strong binding affinities towards μ-opioid receptors with *K*_i_ values of 271 ± 85 nM, 652 ± 159 nM, and 341 ± 29 nM, respectively, indicating their significant analgesic activities [46,47].

It was also recently reported that the methanol extract of the flower buds of *Ch. praecox* showed an inhibitory effect against melanogenesis in ophylline-stimulated B16 melanoma 4A5 cells. A further investigation revealed that the principal alkaloids of (+)-calycanthine (**1**), (−)-chimonanthine (**3**), and (−)-folicanthine (**4**) showed the most potent melanogenesis inhibitory activity, with IC_50_ values of 0.93, 1.4, and 1.8 μM, respectively. Abutin (174 μM), a commercially tyrosinase inhibitor, was used as a positive control. In this paper, (+)-chimonanthine (*ent*-**3**), and *meso*-chimonanthine (**11**) showed cytotoxicity at 10 μM [41]. In another assay, compounds **2**–**4** were screened for the cytotoxicity against a small panel of human cancer lines, showing cytotoxic effects against gastric carcinoma NUGC3 and hepatocarcinoma SNU739 cancer cells with IC_50_ values ranging from 10.3 to 19.7 μM [15].

### 2.3. Biosynthetic Origins

**Scheme 1:**
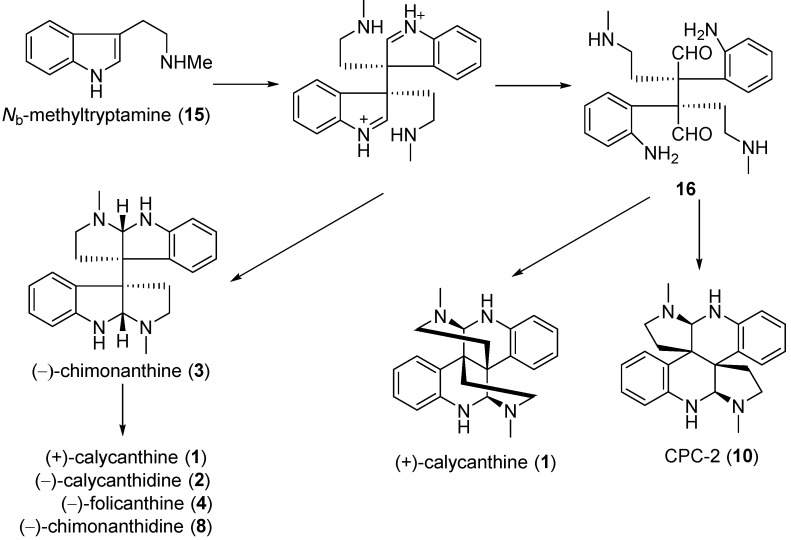
Potential biogenetic pathway of tryptamine-derived dimeric Calycanthaceae alkaloids.

**Scheme 2:**
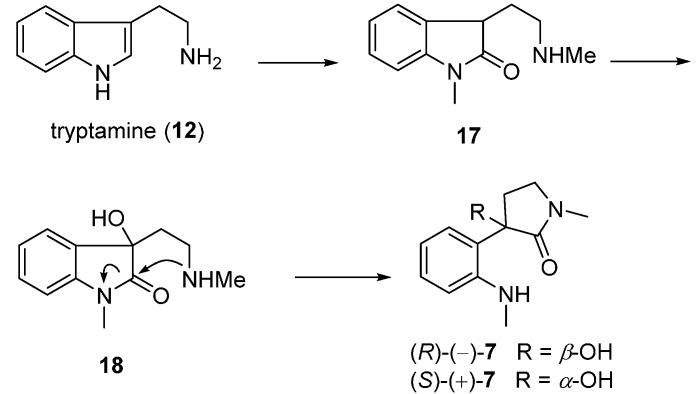
Potential biogenetic pathway of chimonamidine (**7**).

Calycanthine, calycanthidine, chimonanthine, folicanthine, chimonanthidine, and CPC-2 are a series of tryptamine-derived dimeric alkaloids, which were proposed to be originated from *N*_b_-methyltryptamine (**15**). The oxidative dimerization of two molecules of *N*_b_-methyltryptamine forms the key tetraaminodialdehyde intermediate **16**, which undergoes several enzyme-catalyzed reactions and modifications yielding calycanthine and CPC-2 (Scheme 1) [39]. A possible biosynthesis of chimonanmidine (**7**) is shown in Scheme 2. The intermediate **17** was derived from tryptamine (**12**) by oxidation, and subsequently converted into **18** by introduction a hydroxy function at the benzylic position. Chimonanmidine (**7**) was finally produced by the transannulation of the lactam ring of **18** [38,48].

## 3. Total Synthesis of Calycanthaceae Alkaloids

### 3.1. Calycanthines and Chimonanthines

**Figure 2:**
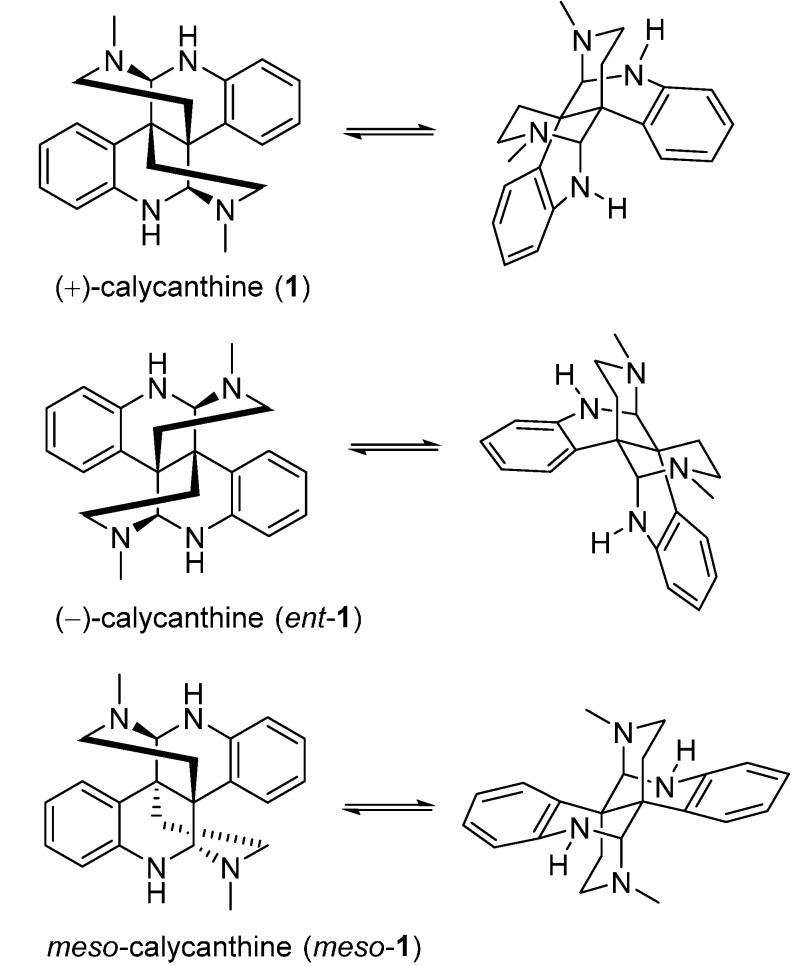
Structures of calycanthines.

The dimeric piperidinoquinoline and pyrrolidinoindoline alkaloids have long been synthetic topics, and efforts have been made on the total synthesis of these two skeletons. Owing to their characteristic *C*_2_-symmetrical bridged bicycles and four chiral centers, three possible calycanthine diastereomers including (+)-calycanthine from Calycanthaceae plants, (−)- and *meso*-calycanthine from *Psychotria forsteriana* [29], were identified (Figure 2). Likewise, the *C*_2_-symmetrical chimonanthines also comprised (±)- and *meso*-chimonanthine. The total syntheses of these alkaloids have been intensively studied for decades. Hino speculated that the structure of 1,1'-dimethyl-3,3'-bis(2-aminoethyl)-3,3'-bioxindole was the key intermediate for the syntheses of calycanthine and (±)-folicanthine [49,50]. Some other groups made synthetic efforts to these bis(pyrroloindoline) scaffold by oxidation dimerization of indole [51] and/or oxindoles derivatives [52,53]. The synthetic challenges were attributed to the two structural features of the dimeric pyrroloindole core, including C3a-C3'a σ bond and its vicinal quaternary stereogenic carbons.

**Scheme 3:**
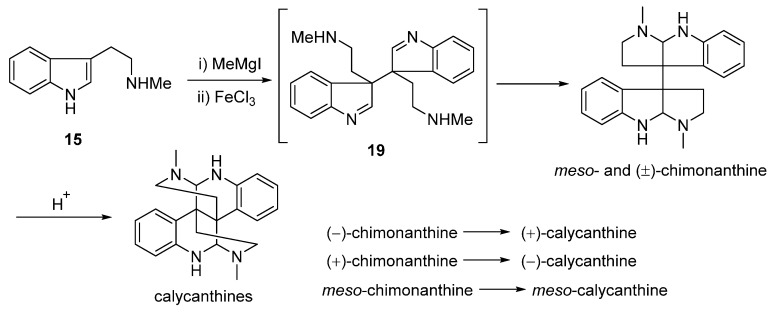
Biomimetic syntheses of chimonanthines and calycanthines proposed by Soctt.

In 1964, Scott’s group established an approach using *N*_b_-methyltryptamine (**15**) as starting material to form the magnesium salt of methyl tryptamine in presence of methyl magnesium iodide (CH_3_MgI). The intermediate was treated with iron(III) chloride to produce the indolenine dimer **19**, which was subsequently transformed to *meso*- and (±)-chimonanthine in one step. Moreover, (±)-calycanthine was accessible by treating (±)-chimonanthine with aqueous acid, demonstrating that structure of **1** was the thermodynamically preferred scaffold (Scheme 3) [54].

**Scheme 4:**
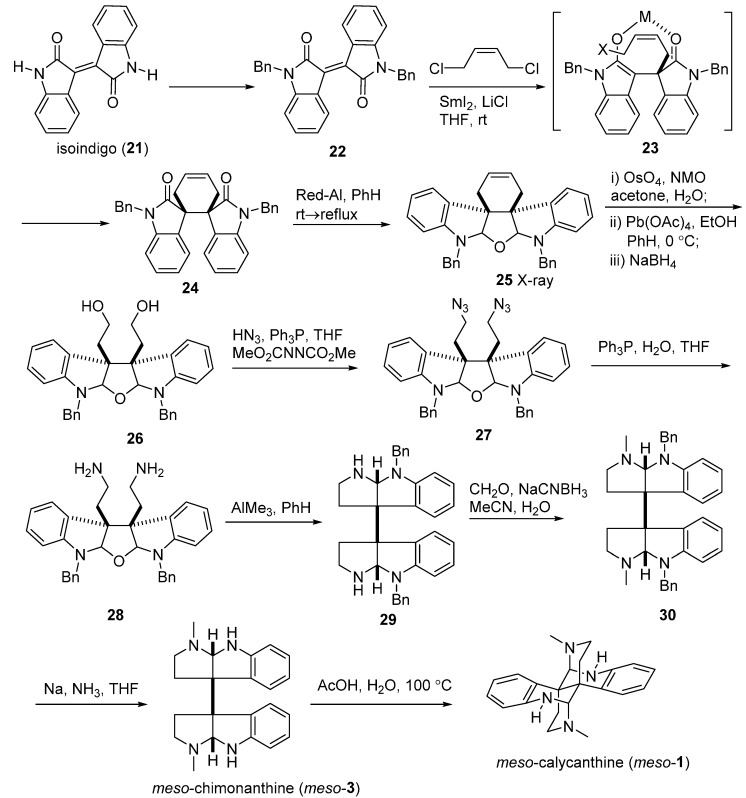
Stereocontrolled syntheses of *meso*-chimonanthine and *meso*-calycanthine by Overman.

**Scheme 5:**
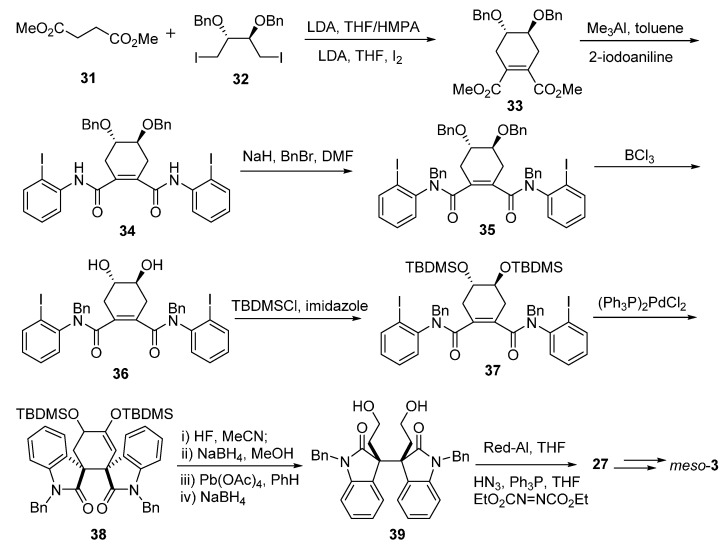
Double Heck cyclizations for *meso*-chimonanthine by Overman.

**Scheme 6:**
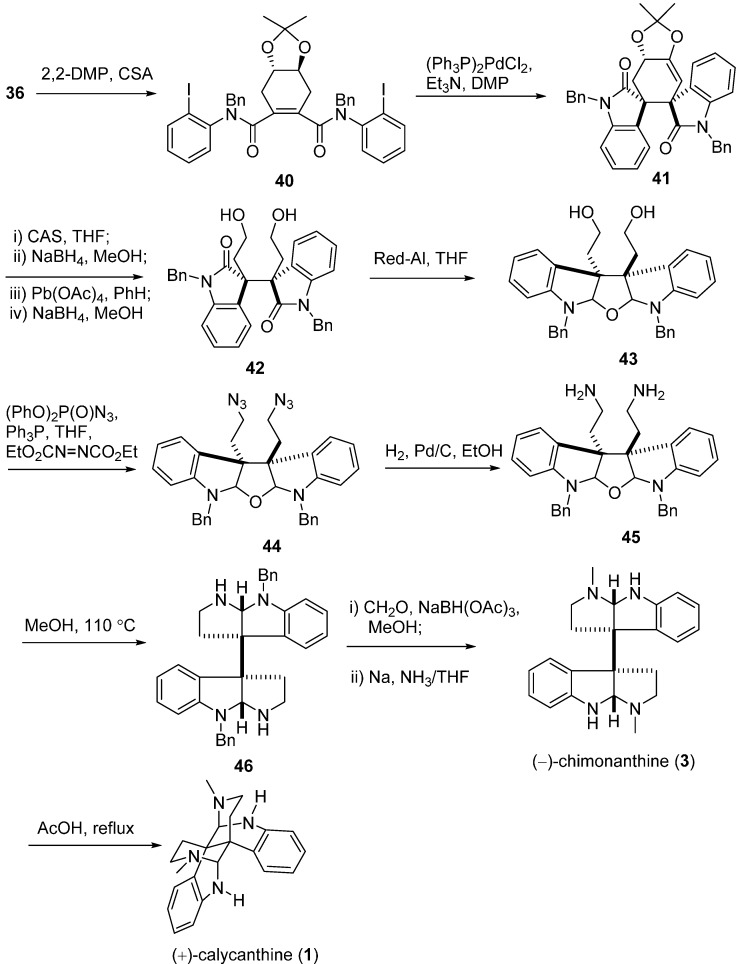
Double Heck cyclizations for (−)-chimonanthine and (+)-calycanthine by Overman.

**Scheme 7:**
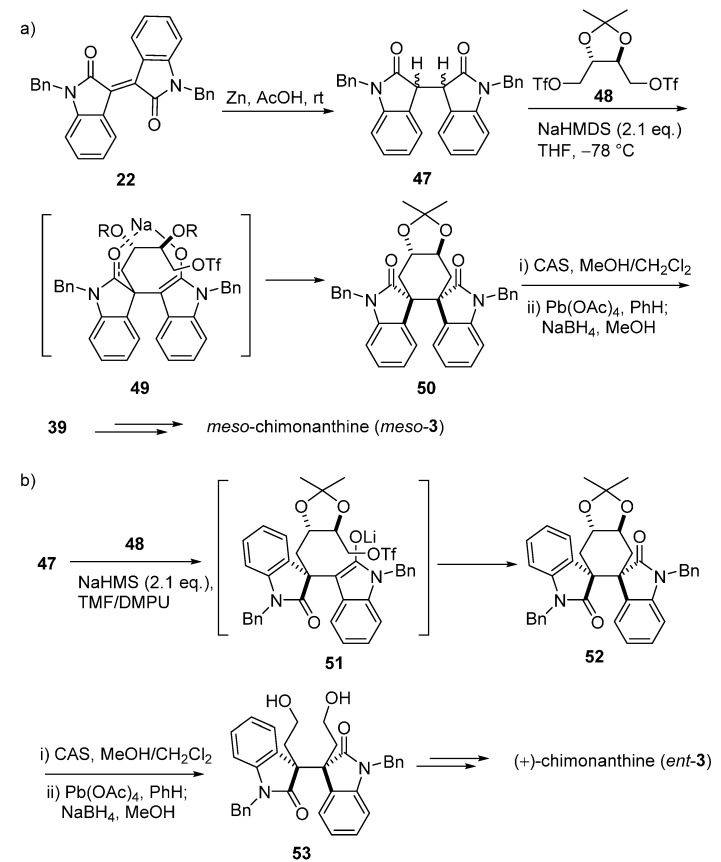
Enantioselective dialkylation approach for *meso*-chimonanthine (**a**) and (+)-chimonanthine (**b**) proposed by Overman.

Overman’s group also proposed systemic syntheses for these Calycanthaceae alkaloids. In 1996, he attempted to stereocontrolly synthesize *meso*-chimonanthine (**11**) and *meso*-calycanthine (*meso*-**1**) via a samarium-mediated reductive dialkylation (Scheme 4). Isoindigo (**21**) was converted to *N*-benzyl derivative **22**, which then generated **24** by the treatment of 2 equiv. of SmI_2_ in the presence of 10 equiv. of LiCl with *cis*-1,4-dicholoro-2-butene. Compound **24** was subsequently transformed into hexacycle **25** with sodium bis(2-methoxyethoxy)aluminum hydride (Red-Al). The cyclohexene of **25** was cleaved to yield **26**, which was immediately reduced to diamine **28** via diazide product **27**. Intermediate **28** was then treated with excess Me_3_Al at room temperature to provide bis(pyrroloindoline) **29**. The desired *meso*-chimonanthine (*meso*-**3**) was produced from **29** by a cascade of methylation and deprotection reactions. The final product *meso*-calycanthine (*meso*-**1**) was obtained by exposure of *meso*-**3** to hot dilute acetic acid [55]. In 1999, a flexible approach to *meso*-chimonanthine (Scheme 5), (−)-chimonanthine, and (+)-calycanthine (Scheme 6) using an intramolecular Heck reaction cascade was present by Overman *et al.* [56]. One year later, they put forward to a highly efficient synthesis of 3a,3a'-bispyrrolidino[2,3-*b*]indolines, a precursor for the *meso*-chimonanthine (Scheme 7a) and (+)-chimonanthine (Scheme 7b). This dialkylation route utilized the reactivities of dienolate (chelated or nonchelated) and the chirality of a tartrate-derived dielectrophile to control the relative configuration and absolute stereochemistry, respectively [35].

**Scheme 8:**
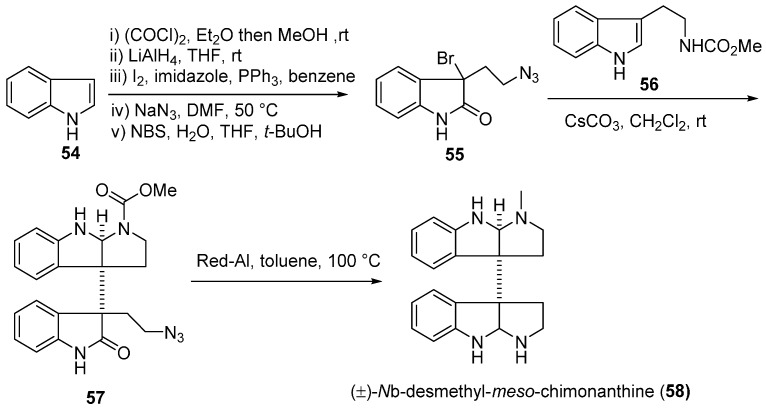
Synthesis of (±)-*N*_b_-desmethyl-*meso*-chimonanthine **(58)** via a tandem [4 + 2]-cycloaddition-cyclisation by Dalko.

In 2006, Dalko’s group provided an elegant synthesis of a homologous compound of *meso*-chimonanthine, *N*_b_-desmethyl-*meso*-chimonanthine. They investigated a tandem [4 + 2]-cycloaddition-cyclisation of a conveniently functionalized bromoxidole (**55**) and tryptamine derivative **56** to construct the *meso*-chimonanthine core in a highly diastereoselective manner (Scheme 8) [57].

**Scheme 9:**
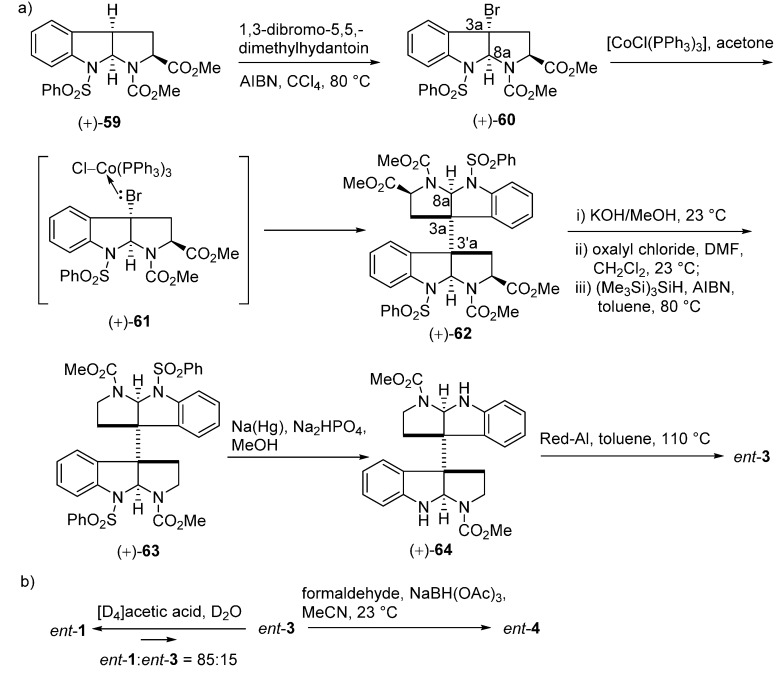
Synthesis of (+)-chimonanthine (*ent*-**3**) (**a**), (+)-folicanthine (*ent*-**4**) and (−)-calycanthine (*ent*-**1**) (**b**) via a reductive Co^I^-promoted homodimerization by Movassaghi.

In 2007, an alternative strategy for the total synthesis of (−)-calycanthine (*ent-***1**) and (+)-chimonanthine (*ent*-**3**) using a reductive Co^I^-promoted dimerization of *endo* bromide (+)-**60** was proposed by Movassaghi. The vital homodimerization requiring a stoichiometric amount of metal catalyst was the key step to secure the vicinal quaternary stereocenters, which was directed by the stereochemistry at the C8a-position of (+)-**60** (Scheme 9a). The further treatment of *ent*-**3** with [D_4_]acetic acid and deuterium oxide provided (−)-calycanthine (*ent*-**1**) (Scheme 9b) [58]. In 2014, Movassaghi’s group also described an enhanced diazene-based method for heterodimerization to enantioselectively synthesize the (−)-calycanthidine (**2**), *meso*-chimonanthine (**11**) and (±)-desmethyl-*meso*-chimonanthine (**58**) [59,60].

**Scheme 10:**
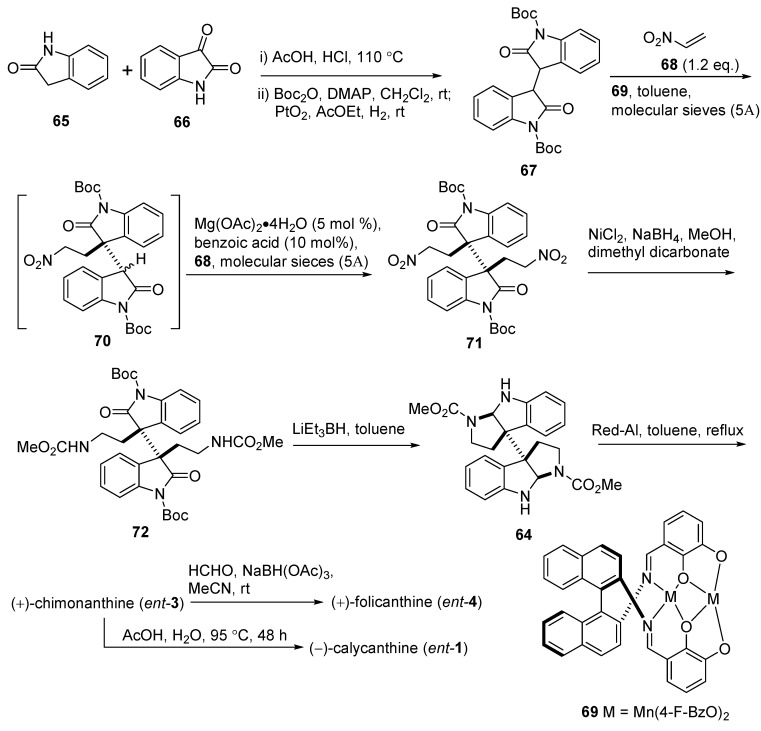
Synthesis of (+)-chimonanthine (*ent*-**3**), (+)-folicanthine (*ent*-**4**) and (−)-calycanthine (*ent*-**1**) via double Michael reaction of bisoxindole by Matsunaga.

In 2012, Kanai and Matsunaga’s group illustrated a straightforward catalytic asymmetric total synthesis to achieve enantioselective (−)-calycanthine (*ent*-**1**) and (+)-chimonanthine (*ent*-**3**) in seven steps (Scheme 10). A one-pot double Michael reaction from 3,3'-bioxindole **67** [61] with the base catalyst Mn(4-F-BzO)_2_/Schiff (**69**) produced the key dialkylated adduct **71** in 69% yield and 95% *ee*. Then the intramolecular dicyclization of **72** was available by the treatment with LiEt_3_BH in toluene [62]. Recently, a new strategy using a double intramolecular carbamoylketene-alkene [2 + 2] cycloaddition for the synthesis of the racemic chimonanthine was accomplished by Shishido’s group (see Chapter 3.2) [63].

### 3.2. Folicanthines

The structures of the folicanthines are also representatives of the dimeric hexahydropyrroloindole alkaloid family. (+)-Folicanthine (*ent*-**4**) could be easily obtained in a quantitative yield from (+)-chimonanthine (*ent*-**3**) by treatment with formaldehyde and sodium triacetoxyborohydride [NaBH(OAc)_3_] (Schemes 9b and scheme10) [58,62]. In striking contrast to the elegant synthetic procedures towards calycanthines and chimonanthines, Gong’s group have developed a highly enantioselective nucleophilic substitution reaction of 3-hydroxyoxindoles with an enecarbamate catalyzed by chiral phosphoric acids.

**Scheme 11:**
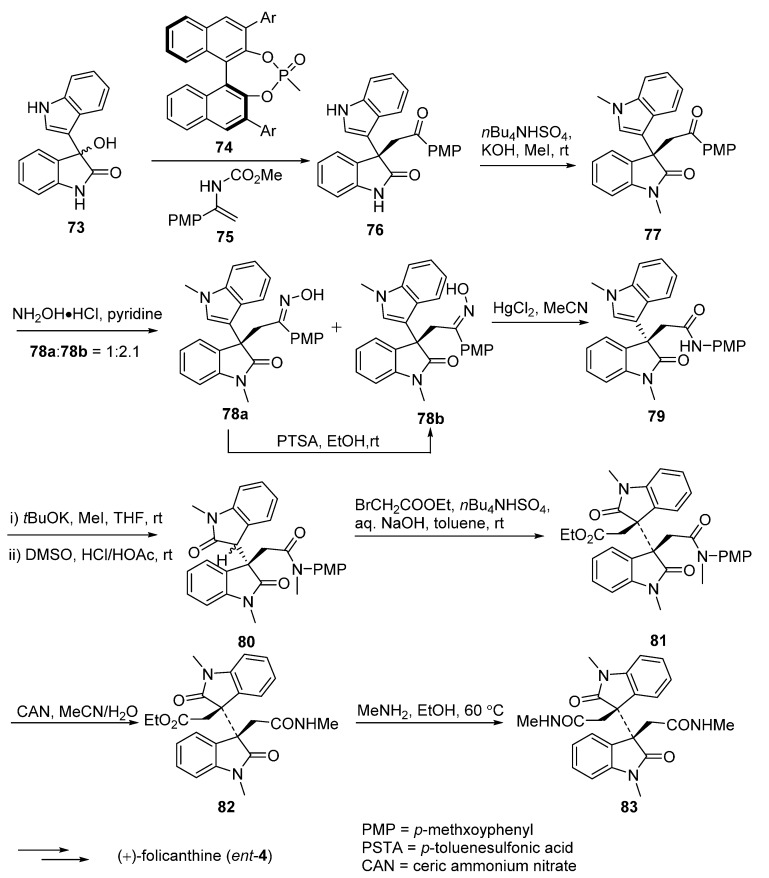
Synthesis of (+)-folicanthine (*ent*-**4**) via asymmetric organocatalytic substitution reaction by Gong.

**Scheme 12:**
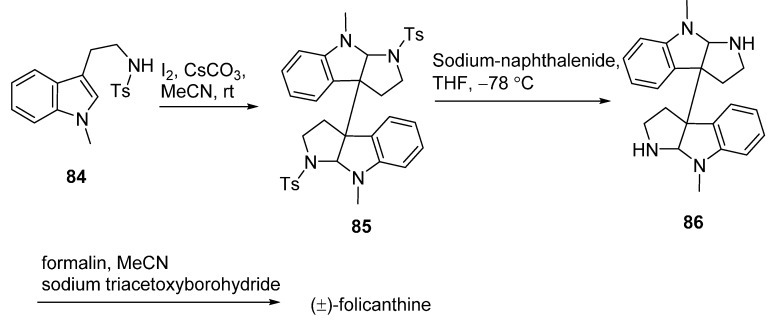
Concise synthesis of (±)-folicanthine by Liang.

**Scheme 13:**
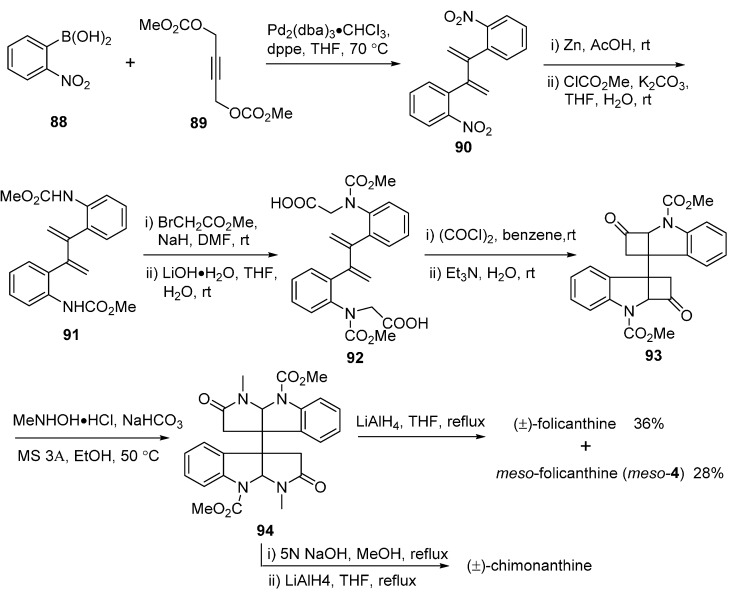
Synthesis of folicanthines and (±)-chimonanthine via a double intramolecular carbamoylketene-alkene [2 + 2] cycloaddition by Shishido.

In Scheme 11, the vital enantioselective substitution reaction of 3-hydroxy-3,3'-bisindolin-2-one (**73**) with **75** was catalyzed by a special chiral phosphoric acids **74** to give **76**. The dimethylation of **76** provided **77**, which was followed with a Beckmann rearrangement to convert the ketones into amide derivatives **78a** and **78b**. A second Beckmann rearrangement of **78b** by the treatment with mercury(II) chloride (Hg_2_Cl) could furnish amide **79**. After introducing a methyl group at the amide nitrogen atom, the product **80** underwent an alkylation to produce key intermediate **81**, which was finally converted to the desired product (+)-folicanthine (*ent*-**4**) after several step sequences [64]. Contemporaneously, Liang’s group described a concise three-step synthesis of (±)-folicanthine using two molecules of 2-(1-methyl-1*H*-indole-3-yl)-*N*-tosylethaneamine (**84**, Scheme 12). The core structure **85** of (±)-folicanthine could be easily obtained by a one-step cyclization-dimerization of substituted tryptophan in high yield. In general, this synthetic route had the advantages of being highly efficient, atom-economic, and metal-free, but it has no enantioselectivity [65]. Recently, Shishido’s group also developed a total synthesis route to access the folicanthines and chimonanthines in racemic form, employing a double intramolecular carboamoylketene-alkene [2 + 2] cycloaddition reaction as the key step (Scheme 13). 2,2'-(Buta-1,3-diene-2,3-diyl)bis(nitrobenzene) (**90**), obtained by a Pd-catalyzed preparation, underwent the key double [2 + 2] cycloaddition to yield bis-carboxylic acid **92**. After a three-step sequence, the mixture of (±)-folicanthine and *meso*-folicanthine (*meso-***4**) was obtained, which was separated by preparative TLC in 36% and 20% yields, respectively [63].

### 3.3. (−)-Idiospermuline (**6**)

**Scheme 14:**
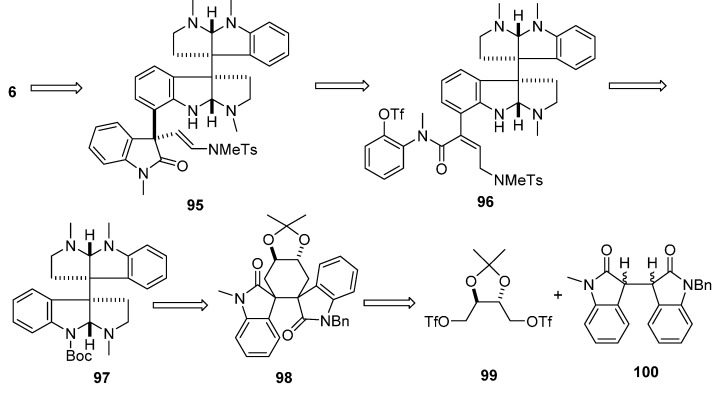
Retrosynthesis of (−)-idiospermuline (**6**).

**Scheme 15:**
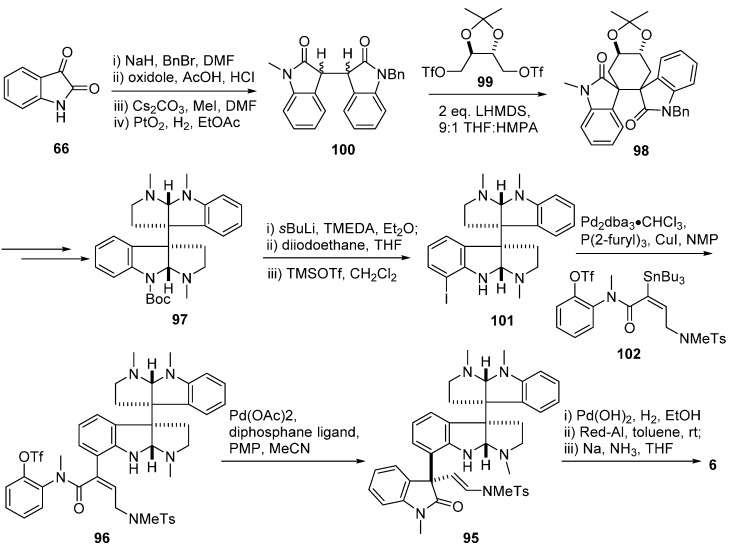
Total synthesis of (−)-idiospermuline (**6**).

(−)-Idiospermuline (**6**), a typical trispyrrolidinoindoline alkaloid, contains three all-carbon quaternary centers, consisting of one (−)-chimonanthine unit and one pyrrolidinoindoline unit with a C3''a–C7' σ bond. Its retrosynthetic analysis was similar to that of hodgkinsines [66] and is presented in Scheme 14, which guided the total enantioselective synthesis of (−)-idiospermuline (Scheme 15). The first step in the synthesis focused on the dimeric pyrrolidinoindoline derivative **97** similar to that of (+)-chimonanthine in Scheme 7. The introduction of the readily available stannyl butenanilide **102** furnished the intermediate **96**. Heck cyclization of **96** treating with chelating diphosphane ligands like bis(1,4-diphenylphosphanyl)butane (dppb) diastereoselectively furnished 3a''*R* precursor **95**. Catalytic hydrogenation of 95 with Pd(OH)_2_ and H_2_, followed by reduction of the carbonyl group with Red-Al in toluene, then Na in NH_3_, provided the desired (−)-idiospermuline (**6**) [67,68,69,70].

### 3.4. Chimonamidines (**7**)

**Scheme 16:**
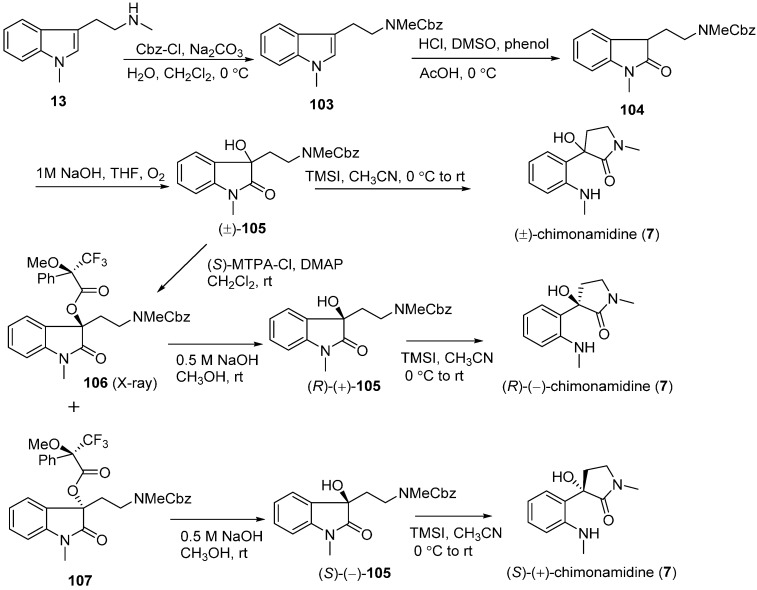
Total synthesis of (±)-chimonamidine (**7**).

Takayama’s group conducted a biomimetic synthesis (Scheme 16) in order to confirm the absolute structure of chimonamidine (**7**). Based on its plausible biogenetic pathway shown in Scheme 2, the precursor *N*_a_,*N*_b_-dimethyltryptamine (**13**) was treated with benzyl chloroformate (Cbz-Cl) and Na_2_CO_3_ to give *N*_a_,*N*_b_-dimethyl-*N*_b_-carbobenzyloxytryptamine (**103**). Oxidation of **103** with dimethyl sulfoxide and hydrochloric acid yielded **104**, which was then converted to the racemic mixture of (±)-hydroxyketones **105** by introduction of a hydroxy group at the benzylic position. The target racemic mixture of (±)-chimonamidine (**7**) was obtained by removing the *N*_b_-Cbz protecting group and forming a new lactam ring under the condition of trimethylsilyl iodide (TMSI). Meanwhile, the chiral synthesis of chimonamidine was also performed by Takayama. After several attempts, a strategy that involved the separation of racemic mixtures by (+)-MTPA chloride and SiO_2_ column chromatography succeeded to yield two diastereomeric esters **106** and **107**. Two more steps that involved the hydrolysis with aqueous alkaline solution and cyclization with TMSI of **106** and **107** were conducted to afford two enantiomerically pure compounds (*R*)-(−)-chimonamidine and (*S*)-(+)-chimonamidine, respectively. A comparison of the optical rotation between synthesized (${\left[\alpha \right]}_{D}^{23}$ = −178 for (*R*)-(−)-**7**; ${\left[\alpha \right]}_{D}^{23}$ = +171 for (*S*)-(+)-**7**) and natural chimonamidine (${\left[\alpha \right]}_{D}^{19}$ = −12.6) came to the conclusion that natural chimonamidine was a mixture slightly enriched with (*R*)-(−)-chimonamidine [38].

### 3.5. Chimonanthidine (**8**)

**Scheme 17:**
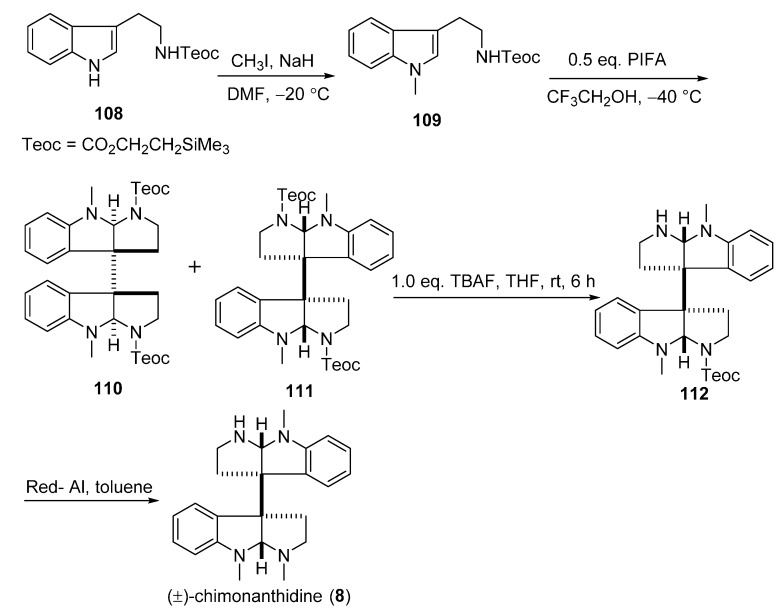
Total synthesis of (±)-chimonanthidine (**8**).

A synthetic approach that uses hypervalent iodine(III) reagents for the dimerization of indole derivatives was developed for the total synthesis of (±)-chimonanthidine (**8**) by Takayama’s group (Scheme 17). The methylation of the known compound **108** with methyl iodide (CH_3_I) and sodium hydride (NaH) in DMF produced *N*_a_-Methyl-*N*_b_-trimethylsilyethoxy-carbonyl (Teoc) tryptamine (**109**), which was then treated with 0.5 equiv. phenyliodine(III) bis(trifluoroacetate) (PIFA) in CF_3_CH_2_OH to yield two dimeric diastereoisomers **110** and **111**. (±)-Chimonanthidine (**8**) was obtained from the monodeprotected amine **112**, which was transformed from **111** by treatment with tetrabutylammonium fluoride (TBAF) in THF [38].

### 3.6. Rac-CPC-1 (Rac-**9**)

**Scheme 18:**
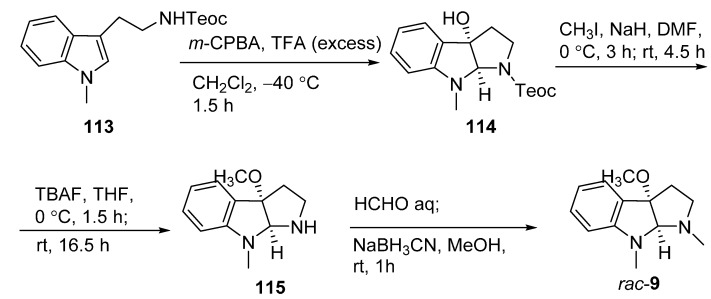
Total synthesis of *rac*-**9**.

**Scheme 19:**
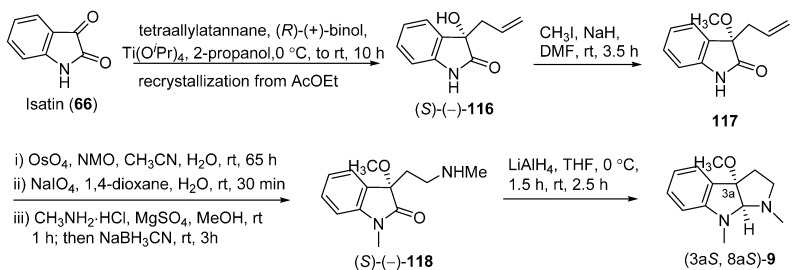
Total synthesis of (3a*S*, 8a*S*)-**9**.

The total synthesis of racemic CPC-1 was initially performed to confirm the structure of CPC-1 (Scheme 18). Compound **113** was treated with *m*-CPBA in the presence of excess trifluoroacetic acid (TFA) in CH_2_Cl_2_ to afford 3a-hydroxypyrrolidinoindoline (**114**). Methylation of the hydroxy group of **114** and then removal of the *N*_b_-Teoc group yielded the intermediate **115**, which was finally treated with formalin and then NaBH_3_CN to give *rac*-**9**. To further establish the absolute configuration of **9**, its chiral total synthesis was conducted (Scheme 19). This synthetic approach started from isatin (**66**), which was treated with (*R*)-(+)-binol, Ti(O*^i^*Pr)_4_, and tetraallylstannane to yield allylated compound **116**. Two recrystallizations of **116** from EtOAc afford enantiomerically pure (*S*)-(−)-**116**. Methylations of *N*_a_ and hydroxy group of (*S*)-(−)-**116** gave the dimethyl compound **117**. The intermediate **117** was treated with OsO_4_ and *N*-methylmorpholine *N*-oxide (NMO) followed by NaIO_4_ to provide the aldehyde, which was directly subjected to reductive amination by condensing with CH_3_NH_2_ and then reduced with NaBH_3_CN to give (*S*)-(−)-**118**. The desired product (3a*S*, 8a*S*)-**9** was obtained by the reductive cyclization of **118**. The optical rotation value of (3a*S*, 8a*S*)-**9** (${\left[\alpha \right]}_{D}^{24}$ = +101) was determined to be opposite of that of **9** (${\left[\alpha \right]}_{D}^{24}$ = −88), indicating the absolute configuration of natural CPC-1 to be 3a*R*,8a*R* [39].

## 4. Conclusions

In conclusion, the Calycanthiaceae plants are rich in promising bioactive dimeric and/or oligomeric piperidinoquinoline and hexahydropyrroloindole alkaloids, which are characterized by unique vicinal quaternary stereocenters. These alkaloids have been a longstanding challenge as total synthetic targets. During the last decades, stereocontrolled total synthetic approaches, such as metal-catalyzed dialkylation, intramolecular double Heck reaction, tandem [4 + 2]-cycloaddition-cyclisation, Co^I^-promoted reductive homodimerization, double Michael reaction, double Beckmann rearrangement, and intramolecular double carbamoylketene-alkene [2 + 2] cycloaddition, have extensively explored. Taken together, these results will keep research on the metabolites of the Calycanthaceae plants as a hot topic for the scientific community in the future.

## List of Abbreviations

Ac: acetyl
Bn: benzyl
Boc: *tert*-butoxycarbonyl
CAN: ceric ammonium nitrate
CAS: camphorsulfonic acid monohydrate
Cbz: carbobenzoxy
*m*-CPBA: *m*-cholorperoxybenzoic acid
dba: *trans,trans*-dibenzylideneacetone
dppe: 1,2-bis(diphenylphosphino)ethane
dppb: bis(1,4-diphenylphosphanyl)butane
DMA: *N,N*-dimethylacetamide
DMF: 2,5-dimethylfuran
DMSO: dimethyl sulfoxide
2,2-DMP: 2,2-dimethioxypropane
DMPU: 1,3-dimethylhexahydro-2-pyrimidinone
HMDS: 1,1,1,3,3,3-hexamethyldisilazane
HMPA: hexamethylphoramide
NaHMDS: sodium hexamethyldisilazide
NMO: 4-methylmorpholine-*N*-oxide
PIFA: phenyliodine(III) bis(trifluoroacetate)
PMP: 1,2,2,6,6-pentamethylpiperidine
PSTA: *p*-toluenesulfonic acid
Red-Al: sodium bis(2-methoxyethoxy)aluminum hydride
TBAF: tertrabutylammonium fluoride
Tf: trifluoromethanesulfonyl
TFA: trifluoroacetic acid
THF: tetrahydrofuran
TMEDA: *N,N,N,N*-tetramethylethylenediamine
TMS: trimethylsilyl
